# Efficient Synthesis and Anti-Tubercular Activity of a Series of Spirocycles: An Exercise in Open Science

**DOI:** 10.1371/journal.pone.0111782

**Published:** 2014-12-10

**Authors:** Katrina A. Badiola, Diana H. Quan, James A. Triccas, Matthew H. Todd

**Affiliations:** 1 School of Chemistry, The University of Sydney, New South Wales, Australia; 2 Microbial Pathogenesis and Immunity Group, Department of Infectious Diseases and Immunology, The University of Sydney, New South Wales, Australia; Univ of Bradford, United Kingdom

## Abstract

Tuberculosis afflicts an estimated 2 billion people worldwide and causes 1.3 million deaths annually. Chemotherapeutic solutions rely on drugs developed many years ago, with only one new therapeutic having been approved in the last 40 years. Given the rise of drug-resistant strains, there is an urgent need for the development of a more robust drug development pipeline. GlaxoSmithKline recently placed the structures and activities of 177 novel anti-tubercular leads in the public domain, as well as the results of ongoing optimisation of some of the series. Since many of the compounds arose from screening campaigns, their provenance was unclear and synthetic routes were in many cases not reported. Here we present the efficient synthesis of several novel analogues of one family of the GSK compounds—termed “Spiros”—using an oxa-Pictet–Spengler reaction. The new compounds are attractive from a medicinal chemistry standpoint and some were potent against the virulent strain, suggesting this class is worthy of further study. The research was carried out using open source methodology, providing the community with full access to all raw experimental data in real time.

## Introduction

Infection by *M. tuberculosis* resulting in symptomatic tuberculosis (TB) can be fatal without treatment. In 2012, TB was responsible for the deaths of 1.3 million people and a further 8.6 million people were infected [1]. Globally, an estimated two billion people carry latent TB and are susceptible to developing active TB. Current first-line treatments include the “short-course-chemotherapy” regime, which involves combinations of rifampicin, isoniazid, pyrazinamide and ethambutol, taken over at least 6 months [2]. These drugs have been in use since the 1960s; the recent FDA approval of bedaquiline [3] makes this drug the first new treatment for TB to be approved in 40 years. The spread of partially- and totally drug resistant strains makes the development of new treatments (preferably targeting new cellular mechanisms) a priority [1].

**Figure 1:**
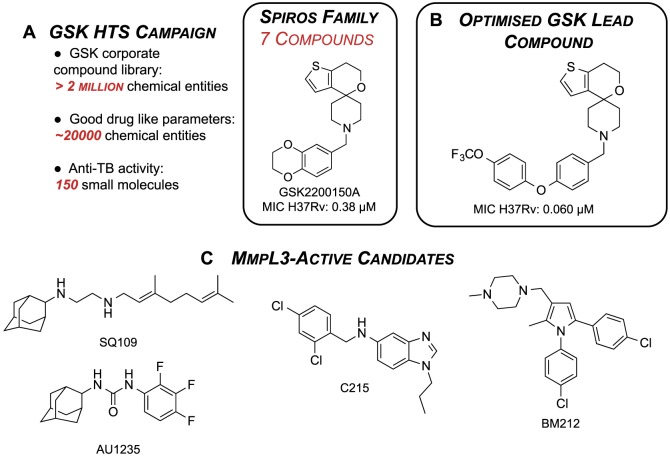
The GSK HTS campaign identified GSK2200150A, which is representative of the GSK Spiros family of anti-TB leads (A). (B) The optimised Spiros analogue developed by GSK [5]. (C) Existing anti-tubercular candidates that have a mode of action that involves MmpL3.

GlaxoSmithKline (GSK) recently published the structures and anti-TB activities of 177 small molecules as part of a deposition of open data [4]. These leads were identified out of a pool of ∼20000 molecules, chosen from the GSK corporate compound collection based on favourable cell permeability and drug-like parameters. Of the 177 leads, seven compounds contained a thiophene spirocycle core; these were termed Spiros by GSK, represented by GSK2200150A (Figure 1).

The members of the Spiros series are excellent starting points for the development of new anti-TB agents. The compounds were identified following a number of screens that evaluated their inhibition of the growth of mycobacteria, cytotoxicity and physical properties. The Spiros appear to affect an essential membrane transport protein (MmpL3) of *M. tuberculosis* [5]. There are no currently-approved drugs that target MmpL3, but four structurally dissimilar compounds (C, Figure 1) have been identified as acting on MmpL3 [6]–[9] as well as a more recent set of indoleamides [10]. In 2012 SQ109 completed a phase IIa clinical trial for pulmonary TB [11]. The Spiros analogues are not overtly similar in structure to these compounds; further investigation into the specific mode of action at MmpL3 is clearly required, but it is a desirable property of any new antitubercular compound that it should have a target different to existing therapeutics.

**Figure 2:**
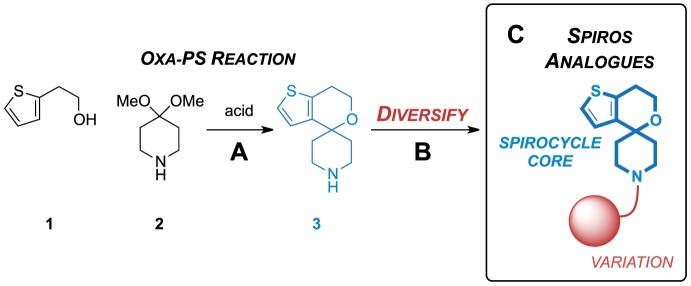
Potential for the rapid synthesis of Spiros analogues via a common 2° amine intermediate 3. (A) An existing oxa-Pictet–Spengler reaction can be used to form the spirocycle core (blue) as the 2° amine **3** [12]. (B) Strategy to diversify from the 2° core **3** to produce Spiros analogues (C) with variation at the piperidine nitrogen (red).

Screening campaigns frequently use commercial libraries that understandably lack synthetic provenance. The synthesis of the secondary amine spirocycle core has been incompletely reported in the patent literature and a synthesis of the GSK hit compound (GSK2200150A) was described in the academic literature but with incomplete data and limited information on analog synthesis. [4]–[5], [12]. We rationalised that Spiros analogues could be rapidly produced by first constructing the core using an oxa-Pictet–Spengler reaction followed by final stage diversification from the secondary amine **3** (Figure 2). This would allow the rapid synthesis of new Spiros analogues in three steps and the faster progression of this series in a hit-to-lead campaign.

We herein describe this work which was conducted using an electronic laboratory notebook on the internet [13] and an open source research philosophy that had shown efficiency gains in the discovery of a synthetic route to a drug used in the treatment of schistosomiasis [14]. The licence governing such work is that the research may be used for any purpose, including for financial gain, provided the project is cited. The relevant laboratory notebook, containing a browseable snapshot of the experiments and all the data for the period April–August 2013, has been deposited online [15]. Data for remaining experiments (period late August–December 2013) are included in Spreadsheet S1 rather than the electronic laboratory notebook due to a local technical difficulty at the time these data were collected.

## Results and Discussion

### Synthesis of the Spiros Analogues

**Figure 3:**
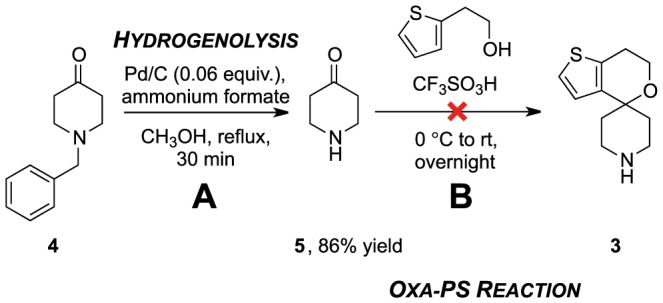
Synthesis of the core 70 was attempted using conditions adapted from the literature [12]. (A) *N* -Debenzylation of **4** was achieved under palladium-catalysed transfer hydrogenation conditions [17]. (B) Reaction of the ketone **5** with thiopheneethanol **1** in the presence of strong Brønsted acids did not produce the expected spirocycle **3**.

Our first synthetic approach was the use of compound **5** to make **3** with an oxa-Pictet Spengler reaction (Figure 3). We adapted the patent procedure (A, Figure 2) [12] by using 4-piperidone **5**, instead of the corresponding ketal **2**, since substitution of the ketal for the ketone in the oxa-Pictet–Spengler reaction should have a limited effect the reaction outcome [16]. The ketone is commercially available but was easily prepared from commercial 4-benzylpiperidinone **4** (A, Figure 3) [17] a compound that was itself later used. However, the harsh conditions of the cyclization (excess triflic acid) resulted in the formation of intractable mixtures.

Several reaction variables were explored to promote cyclisation, such as reducing the amount of acid, using the less acidic methanesulfonic acid [18]–[19], introducing a non-polar solvent (toluene or dioxane) and heating the reaction. These efforts resulted in either partial recovery of the thiophene starting material along with a complex product mixture or decomposition and the formation of polar compounds that were not identified.

**Figure 4:**
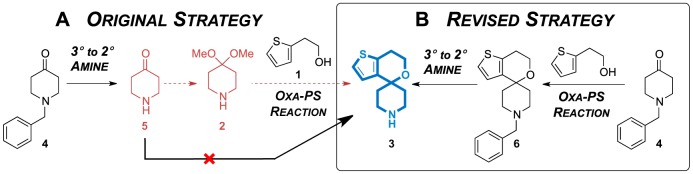
Re-evaluating the route to the 2° amine core 3 (blue). (A) The attempted route based on the patent literature procedure [12]. The dotted lines represent the additional step (red) required to attempt cyclisation strictly under the patent conditions. (B) The revised strategy: cyclise to give **6** followed by debenzylation to give the 2° amine **3** (blue).

The ketal **2** may therefore be crucial in this transformation where precise tuning of reactivity and conditions is necessary to promote conversion but not decomposition. However, formation of this ketal would unfavourably introduce another step in the synthesis (A, red, Figure 4). We therefore devised an alternate strategy for accessing the secondary amine **3**: carrying out the oxa-Pictet–Spengler reaction using **4** prior to removal of the *N*-benzyl group (B).

**Figure 5:**
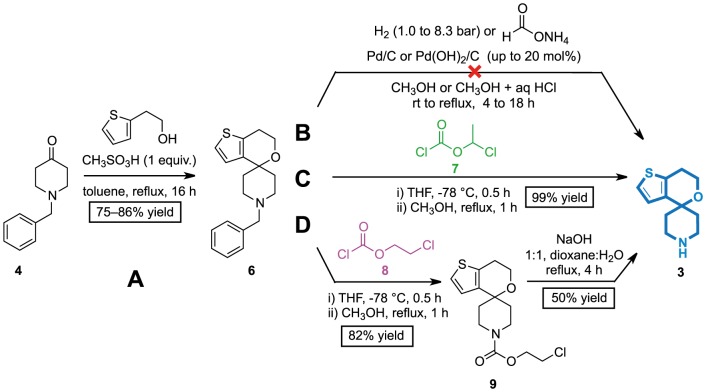
Executing the revised strategy towards the synthesis of spirocycle core as the 2° amine 3. (A) The acid-mediated cyclisation. (B) Attempts to synthesise the 2° amine using catalytic hydrogenolysis. (C) 1-chloroethyl chloroformate was effective at producing the secondary amine **3**. (D) 2-chloroethyl chloroformate resulted in incomplete deprotection of **6**.

The first step of the revised strategy proved effective; the *N*-benzylated core (**6**) was obtained in consistently good yield over a number of repeats (A, Figure 5). The reaction was promoted by 1.5 equivalents of methanesulfonic acid instead of triflic acid, the former being easier to handle. To counter the reduced acidity, the reaction temperature was increased. Lowering the acid loading or reaction time gave a mixture of the product **6** and starting material; the ketone **4** was inseparable from the product **6** in the post-reaction workup or by flash chromatography. The methanesulfonic acid-mediated reaction initially carried out was the most effective at cleanly obtaining the desired spirocycle **6**.

The next step was the removal of the *N*-benzyl group from **6** to give the secondary amine **3** (B to D, Figure 5). The *N*-benzylated spirocycle **6** was stable to a variety of common palladium-catalysed hydrogenolysis conditions (B). The transfer hydrogenation conditions used in the preparation of 4-piperidinone **5** were ineffective; starting material **6** was recovered. Subsequent attempts were made using different combinations of pressure (hydrogen gas up to 8.3 bar), transfer hydrogenation and extended reaction times. In all attempts, starting material was recovered with minimal loss of material.

We turned instead to debenzylation conditions mediated by 1-chloroethyl chloroformate **7** (green), which gave the expected secondary amine **3** (blue) in excellent yield (C, Figure 5). The debenzylation proceeds presumably *via* a carbamate intermediate following the reaction of the starting material **6** with the chloroformate **7** and loss of benzyl chloride [20]–[21]. Subsequent decarboxylation, promoted by the excess of methanol and reflux conditions, produced the desired secondary amine **3** [20]–[21]. Isolation of the carbamate **9** when 2-chloroethyl chloroformate **8** was used is consistent with the proposed mechanism (D); the initial *N*-debenzylation step would be unaffected given the similar reactivity of the chloroformate functional groups, but methanol attack on the secondary carbon to lose the β-chloride would be less likely than attack on the tertiary carbon to lose the α-chloride. The secondary amine **3** was obtained, ready for final stage diversification in ∼84% yield over two steps.

**Figure 6:**
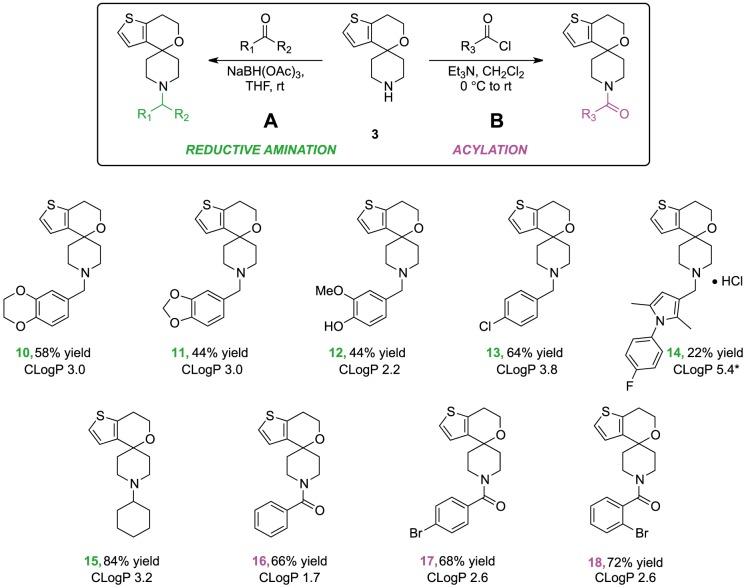
Diversification in the final step. The conditions for reductive amination (A) and acylation (B) of 3 to produce the final Spiros analogs **10–18** the yields reported are of pure material isolated following flash chromatography on silica.

Diversification from the secondary amine **3** using reductive amination and acylating conditions enabled the rapid synthesis of a variety of compounds in moderate to good yield (Figure 6). The sodium triacetoxyborohydride-mediated reductive amination procedure was adapted from the literature [22]–[23]. Acylation of the amine **3** was achieved using aroyl chlorides. All candidate compounds were designed to exhibit acceptable calculated logP values.

**Figure 7:**
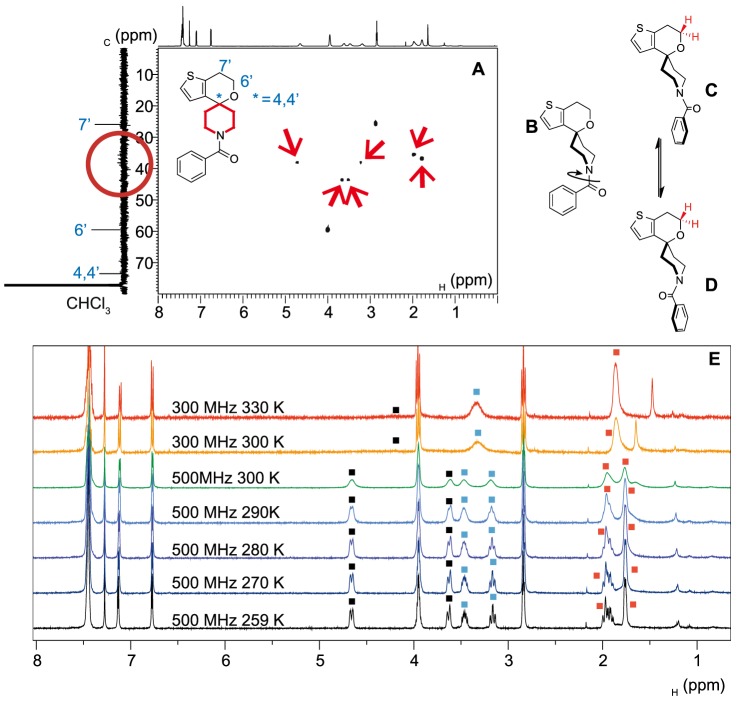
The piperidine nitrogen signals of the acylated products can be visualised by HSQC experiments. The ^1^H-NMR and ^13^C{^1^H}-NMR spectra respectively displayed on the x- and y-axes of the HSQC spectra are projections of the corresponding one-dimensional NMR experiments and are displayed for clarity. (A) The aliphatic region of the ^1^H–^13^C{^1^H} HSQC spectrum of **16**; the red arrows indicate the ^1^H signals corresponding to the piperidine protons (attached to the red ring of the structure). The corresponding piperidine carbon signals on the y-axis (circled red) are unclear in the ^13^C{^1^H} spectrum. (B) Rotation around the amide bond generates chiral rotamers (C and D) dictated by the π-system of the benzamide, which makes the methylene protons in the oxygen-containing ring diastereotopic, (E) Variable magnetic field temperature and NMR experiments showing coalescence of methylene signals for compound **16**. At lower temperature the diastereotopic oxygen-containing ring protons (E) exhibited higher order coupling, arising from the individual rotamers (Figure S39) and the ^13^C{^1^H} NMR spectrum exhibited complete resolution of the piperidine ring carbon signals (Figure S40). Raw data may be found in Dataset S1.

The acylation products **16** to **18** and the arylpyrrole **14** exhibited convoluted NMR spectra. The ^13^C{^1^H}-NMR spectra of **16** to **18** contained the signals expected from the carbon atoms in the carbonyl groups (165 to 175 ppm in CDCl_3_) indicating formation of the amide bond, but ^1^H–^13^C{^1^H} Heteronuclear Single Quantum Correlation (HSQC) spectroscopy was required to elucidate the piperidine ^13^C{^1^H} region (A, Figure 7); the broad ^1^H signals correlated to the aliphatic region of the ^13^C{^1^H} spectrum (B) were consistent with the piperidine ring protons and all environments were accounted for. The broad signals observed for the methylene protons on the oxygen-containing ring in the compounds containing an exocyclic amide bond (i.e. compounds **16–18**) most likely arise from the chirality exhibited by the individual rotamers (C and D), which, though in dynamic equilibrium *via* an achiral intermediate (B), make the protons attached to these carbons diastereotopic. Additional experiments were carried out on the acylated product **16**, which showed temperature and magnetic field dependence (E) consistent with rapid rotation of the amide bond; at higher fields or lower temperatures peaks for the individual rotamers, and their more convoluted splitting patterns, became clear.

### Activity of the Spiros Analogues

The activities of the compounds containing the spirocycle core (**3, 6, 9–18**) were determined against the virulent *M.tuberculosis* strain (H37Rv) (Table 1). Initially, *M. tuberculosis* H37Rv was exposed to a single compound dose of 100 µM for 7 days, and survival was determined in comparison to vehicle-treated bacterial cells using a Resazurin microtiter assay of growth inhibition [24]. The potency of compounds displaying activity at 100 µM was determined by calculating the concentration of drug inhibiting 50% of bacterial growth (IC_50_). Excellent inhibitory activity against H37Rv for compounds containing the *N*-benzyl/pyrrole-type centres (**6**, **10**, **11**, **13** and **14**) was observed other than for **12**; the cyclohexyl-containing spirocycle **1**, the secondary amine **3** and the *N*-acyl-type spirocycles (**9, 16–18**) were inactive. The presence of the *N*-CH_2_-Ar group appeared to be a necessary but not sufficient condition for significant activity against H37Rv, but amide-type functionality was detrimental to the potency of the molecules. Novel compounds **13** and **14** were the most potent with activity comparable to the Spiros anti-TB compounds identified and developed by GSK [4]–[5].

**Table 1:** Anti-tubercular activity of the synthesised Spiros analogues.

Entry	Compound	CLogP	H37Rv IC_50_ (µM)	THP IC_5_ (µM)
1	**3**	0.83	>100	n/a
2	**6**	3.1	5	200
3	**9**	2.1	>100	n/a
4	**10**	3.0	1.25	100
5	**11**	3.0	10	>200
6	**12**	2.2	>100	n/a
7	**13**	3.8	1.25	50
8	**14**	5.4*	1.25	6.3
9	**15**	3.2	>100	n/a
10	**16**	1.7	>100	n/a
11	**17**	2.6	>100	n/a
12	**18**	2.6	>100	n/a

Compounds with a minimum inhibitory concentration >25000 nM were not tested in the inhibition assay of starting concentration 2 mM. The CLogP were calculated using ChemBioDraw Ultra 12.0.3 and the values are a guide only. CLogP values of rifampicin were also calculated using the same method for consistency.

*The CLogP value was calculated for the free compound.

Toxicity of compounds displaying anti-H37Rv activity was assessed using the human monocytic cell line THP1 (Table 1). The IC_50_ against THP was calculated and was compared to the IC_50_ calculated against *M. tuberculosis* H37Rv. Compounds **6**, **10**, **11** and **13** displayed THP1 toxicity at relatively high concentrations (>50 µM), suggesting potential for the future development of these compounds. Compounds **6** and **11** were less toxic than the original GSK structure (**10**) yet were also less potent against H37Rv. Compound **14** was highly active against H37Rv however was toxic against THP1 in the low µM range.

## Conclusion

A three-step synthesis of new TB drug leads is reported that will provide rapid access to potent compounds that may be used in a future assessment of this series. This should both aid in the synthesis of existing analogues for examination of their pharmacokinetic properties and the synthesis of diverse new analogues in this series to optimise potency and drug likeness as well as to further mechanism of action studies. Future participants in such efforts are encouraged to adopt the open platform that has already been developed for full data sharing and collaboration.

## Materials and Methods

### General Chemistry

General synthetic and analytical methods are detailed in Text S1. Raw NMR data for all compounds are available from The University of Sydney eScholarship Repository [25]. The open electronic laboratory notebook for experiments carried out April–August 2013 is available from The University of Sydney eScholarship Repository [15]; all other experiments are summarised in Spreadsheet S1.

### 6′,7′-Dihydrospiro[piperidine-4,4′-thieno[3,2-*c*]pyran] 3

This compound exists in the literature but no data are reported [22]. To a stirred solution of **6** (2.3 g, 7.7 mmol, 1 equiv.) in anhydrous THF (200 mL) under argon at −78°C was added 1-chloroethyl chloroformate (1.6 mL, 15 mmol, 2 equiv.). The reaction mixture was stirred for 30 min then allowed to warm to rt. THF was removed under reduced pressure leaving a residual volume of ∼10 mL which was diluted with methanol (200 mL) and heated at reflux for 20 min. The now clear brown solution was concentrated under reduced pressure to give the crude product as a brown foam (2.8 g). The crude material was purified by flash chromatography on silica (5∶100∶0.5, CH_3_OH∶CH_2_Cl_2_∶NH_4_OH, v/v to 10∶100∶1, CH_3_OH∶CH_2_Cl_2_∶NH_4_OH, v/v) to give *the title compound* as a pale brown solid (1.6 g, 99%); mp 86–88°C; ν_max_ (film)/cm^−1^ 2953, 2923, 1072, 1050, 741, 654; δ_H_ (500 MHz; CDCl_3_) 7.06 (d, *J* = 5.2 Hz, 1 *H*), 6.79 (d, *J* = 5.2 Hz, 1 *H*), 3.93 (t_app_, *J* = 5.4 Hz, 2 *H*), 3.06–3.01 (m, 2 *H*), 2.90–2.87 (m, 2 *H*), 2.82 (t_app_, *J = *5.3 Hz, 2 *H*), 1.85–1.78 (m, 4 *H*), 1.66 (brs, 1 *H*) (Figure S1); δ_C_ (126 MHz; CDCl_3_) 141.6, 132.5, 124.5, 122.4, 73.9, 59.1, 42.2, 37.0, 26.0 (Figure S2); **^1^**H –^13^C HSQC (Figure S3) and ^1^H –^13^C{^1^H} HMBC (Figure S4) spectra also supplied; Anal. Calcd. for C_11_H_15_NOS: C, 63.12; H, 7.22; N, 6.69. Found: C, 63.46; H, 7.56; N, 6.61.

### 1-Benzyl-6′,7′-dihydrospiro[piperidine-4,4′-thieno[3,2-*c*]pyran] 6

To a vigorously stirred solution of thiopheneethanol (0.50 mL, 4.5 mmol, 1 equiv.) and 1-benzyl-4-piperidinone (0.80 mL, 4.5 mmol, 1 equiv.) in toluene (50 mL) was added methanesulfonic acid (0.29 mL, 4.5 mmol, 1 equiv.). The reaction mixture was heated at reflux (oil bath at 130°C) for 16 h and allowed to cool to rt. The mixture was diluted with ethyl acetate (50 mL) and washed with saturated NaHCO_3_ (30 mL). The aqueous fraction was extracted with ethyl acetate (2×30 mL), further basified with solid sodium hydroxide (4.0 g) and extracted with ethyl acetate (2×30 mL). The combined organic fractions were dried (Na_2_SO_4_) and concentrated under reduced pressure to give the crude product as a dark brown oil (1.7 g). The crude material was purified by flash chromatography on silica (10–50% ethyl acetate/hexanes) to give *the title compound* as a pale yellow oil (1.2 g, 86%); ν_max_ (film)/cm^−1^ 2936, 2813, 1075, 854, 672; δ_H_ (500 MHz; CDCl_3_) 7.37–7.31 (m, 4 *H*), 7.27–7.24 (m, 1 *H*), 7.06 (d, *J* = 5.2 Hz, 1 *H*), 6.82 (d, *J* = 5.2 Hz, 1 *H*), 3.93 (t_app_, *J = *5.4 Hz, 2 *H*), 3.56 (s, 2 *H*), 2.82 (t_app_, *J = *5.4 Hz, 2 *H*), 2.74–2.71 (m, 2 *H*), 2.43–2.38 (m, 2 *H*), 2.01–1.95 (m, 2 *H*), 1.86–1.82 (m, 2 *H*) (Figure S5); δ_C_ (126 MHz; CDCl_3_) 141.3, 138.6, 132.7, 129.4, 128.3, 127.1, 124.5, 122.3, 73.3, 63.5, 59.1, 49.2, 36.2, 26.0 (Figure S6); **^1^**H –^13^C HSQC (Figure S7) and ^1^H –^13^C{^1^H} HMBC (Figure S8) spectra also supplied; HRMS (ESI) 300.14161 ([M+H]^+^), calcd. for C_18_H_22_NOS^+^ 300.14166.

### Reductive amination: general procedure 1

This procedure was adapted from the literature [20] [22]. Compounds **10–15** were prepared using this method. To a stirred solution of secondary amine **3** (1 equiv.) and the appropriate aldehyde or ketone (1.1 equiv.) in anhydrous dichloromethane (to 50 mM of 70) was added sodium triacetoxyborohydride (1.5 equiv.). The mixture was stirred at rt under argon for 18–24 h then quenched by pouring over saturated NaHCO_3_ solution. The aqueous phase was extracted with dichloromethane (2 times). The combined organic fractions were dried (Na_2_SO_4_) and concentrated under reduced pressure. The crude product was purified by flash chromatography on silica to give the corresponding tertiary amine product.

### Acylation: general procedure 2

Compounds **16–18** were prepared using this method. A stirred solution of secondary amine **3** (1 equiv.) and triethylamine (2 equiv.) in anhydrous dichloromethane (to 0.2 M of 70) under argon was cooled in a brine ice bath. The appropriate acid chloride (1 equiv.)—either benzoyl chloride, 2-bromobenzoyl chloride or 4-bromobenzoyl chloride—was slowly added. The mixture was stirred at temperature for 15 min, slowly allowed to warm to rt and stirred for a further for 12–15 h. The reaction was quenched by pouring over saturated NaHCO_3_ solution. The aqueous phase was extracted with dichloromethane (3 times). The combined organic fractions were dried (Na_2_SO_4_) and concentrated under reduced pressure. The crude product was purified by flash chromatography on silica to give the corresponding amide product.

### 2-Chloroethyl 6′,7′-dihydrospiro[piperidine-4,4′-thieno[3,2-*c*]pyran]-1- carboxylate 9

This procedure was adapted from the literature [22]. To a stirred solution of **3** (0.40 g, 1.4 mmol, 1 equiv.) in anhydrous THF (35 mL) under argon at −78°C was added 2-chloroethyl chloroformate (0.18 mL, 1.7 mmol, 1.3 equiv.). The reaction mixture was stirred at −78°C for 30 min then allowed to warm to rt. The solvent was removed under reduced pressure and the brownish residue was suspended in methanol (40 mL) and heated at reflux for 1 h. The now clear brown solution was concentrated under reduced pressure to give the crude product as a yellow oil (∼0.5 g). The crude material was purified by flash chromatography on silica (10% ethyl acetate/CH_2_Cl_2_) to *give the title compound* as a colourless oil that solidified when taken to 4°C (0.35 g, 82%); mp 57–60°C; ν_max_ (film)/cm^−1^ 2953, 2923, 1072, 1050, 741, 654; δ_H_ (500 MHz; CDCl_3_) 7.08 (d, *J = *5.2 Hz, 1 *H*), 6.72 (d, *J = *5.2 Hz, 1 *H*), 4.36 (t_app_, *J = *5.7 Hz, 2 *H*), 4.04 (brs, 2 *H*), 3.94 (t_app_, *J = *5.4 Hz, 2 *H*), 3.71 (t, *J = *5.6 Hz, 1 *H*), 3.21 (s, 1 *H*), 2.83 (t, *J = *5.4 Hz, 1 *H*), 1.84–1.81 (m, 1 *H*) (Figure S9); δ_C_ (126 MHz; CDCl_3_) 154.9, 140.3, 132.9, 124.1, 122.7, 73.2, 65.0, 59.4, 42.5, 39.9, 25.9 (Figure S10); HRMS (ESI) 280.10010 ([M−Cl]^+^) calcd. for C_14_H_18_NO_3_S^+^ 280.10019; **^1^**H –^13^C HSQC (Figure S11) and ^1^H –^13^C{^1^H} HMBC (Figure S12) spectra also supplied; HRMS (ESI) 338.05867 ([M+Na]^+^), calcd. for C_14_H_18_ClNO_3_SNa^+^ 338.05881; Anal. Calcd. for C_14_H_18_ClNO_3_S: C, 53.24; H, 5.74; N, 4.44. Found: C, 53.21; H, 5.77; N, 4.40. ^13^C{^1^H}-NMR signals are missing or obscured due to rotamers around the carbamate.

### 1-((2,3-Dihydrobenzo[*b*][1], [4]dioxin-6-yl)methyl)-6′,7′-dihydrospiro[piperidine- 4,4′-thieno[3,2-*c*]pyran] 10

Prepared according to general procedure 1. Following purification by flash chromatography (1∶100∶0.1, CH_3_OH∶CHCl_3_∶NH_4_OH), *the title compound* was obtained as a colourless oil that solidified to a colourless solid when taken to -20°C (52 mg, 58%); mp 106–109°C; ν_max_ (film)/cm^−1^ 2926, 2813, 1505, 1068, 886, 648; δ_H_ (500 MHz; CDCl_3_) 7.06 (d, *J = *5.1 Hz, 1 *H*), 6.88 (s_app_, 1 *H*), 6.84–6.79 (m, 3 *H*), 4.25 (s, 4 *H*), 3.92 (t_app_, *J = *5.3 Hz, 2 *H*), 3.45 (s, 2 *H*), 2.82 (t_app_, *J = *5.3 Hz, 2 *H*), 2.71 (brd_app_, *J = *11.2 Hz, 2 *H*), 2.37 (t_app_, *J = *11.9 Hz, 2 *H*), 1.97 (td_app_, *J = *13.2, 4.0 Hz, 2 *H*), 1.84 (d, *J = *13.0 Hz, 2 *H*) (Figure S13); δ_C_ (126 MHz; CDCl_3_) 143.4, 142.7, 141.4, 132.7, 132.0, 124.6, 122.40, 122.30, 118.1, 117.0, 73.4, 64.51, 64.49, 62.9, 59.1, 49.1, 36.3, 26.0 (Figure S14); **^1^**H –^13^C HSQC (Figure S15) and ^1^H –^13^C{^1^H} HMBC (Figure S16) spectra also supplied; HRMS (ESI) 358.14712 ([M+H]^+^), calcd. for C_20_H_24_NO_3_S^+^ 358.14714; Anal. Calcd. for C_20_H_23_NO_3_S: C, 67.20; H, 6.49; N, 3.92. Found: C, 66.55; H, 6.54; N, 3.88. Discrepancy in CHN analysis noted, but data consistent with 4M+ H_2_O, i.e. calcd. for C_80_H_94_N_4_O_13_S_4_ 66.36; H, 6.54; N, 3.87.

### 1-(Benzo[*d*] [1], [3]dioxin-5-ylmethyl)-6′,7′-dihydrospiro[piperidine- 4,4′-thieno [3,2-*c*]pyran] 11

Prepared according to general procedure 1. Following purification by flash chromatography (2∶100∶0.2, CH_3_OH∶CH_2_Cl_2_∶NH_4_OH), *the title compound* was obtained as a colourless oil that solidified to a colourless solid when taken to 4°C (37 mg, 44%); mp 81–84°C; ν_max_ (film)/cm^−1^ 2923, 2813, 1501, 1487, 1440, 1242, 1074, 1036, 934, 853, 648; δ_H_ (500 MHz; CDCl_3_) 7.06 (d, *J = *5.2 Hz, 1 *H*), 6.89 (d_app_, *J = *1.0 Hz, 1 *H*), 6.81 (d, *J* = 5.2 Hz, 1 *H*), 6.78 (dd, *J = *8.0, 1.0 Hz, 1 *H*), 6.75 (d_app_, *J = *8.0 Hz, 1 *H*), 3.92 (t_app_, *J = *5.4 Hz, 2 *H*), 3.47 (s, 2 *H*), 2.82 (t_app_, *J = *5.4 Hz, 2 *H*), 2.72–2.70 (m, 2 *H*), 2.40–2.35 (m, 2 *H*), 2.00–1.94 (m, 2 *H*), 1.86–1.83 (m, 2 *H*) (Figure S17); δ_C_ (126 MHz; CDCl_3_) 147.7, 146.7, 141.3, 132.73, 132.56, 124.5, 122.48, 122.33, 109.8, 108.0, 101.0, 73.4, 63.3, 59.1, 49.1, 36.3, 26.0 (Figure S18); HRMS (ESI) 344.13146 ([M+H]^+^), calcd. for C_19_H_22_NO_3_S^+^ 344.13149; Anal. Calcd. for C_19_H_21_NO_3_S: C, 66.45; H, 6.16; N, 4.08. Found: C, 66.59; H, 6.26; N, 3.98.

### 4-((6′,7′-Dihydrospiro[piperidine-4,4′-thieno[3,2-*c*]pyran]-1-yl) (2-methoxyphenol) 12

Prepared according to general procedure 1. Following purification by flash chromatography (2∶100∶0.1, CH_3_OH∶CH_2_Cl_2_∶NH_4_OH), *the title compound* was obtained as a colourless solid (41 mg, 44%); mp 170–172°C; ν_max_ (film)/cm^−1^ 2927, 1517, 1277, 1265, 1071, 800, 732; δ_H_ (500 MHz; CDCl_3_) 7.04 (d, *J = *5.2 Hz, 1 *H*), 6.90 (s_app_, 1 *H*), 6.83–6.77 (m, 2+1 *H*), 3.92 (t_app_, *J = *5.4 Hz, 2 *H*), 3.83 (s, 3 *H*), 3.50 (s, 2 *H*), 2.82 (t_app_, *J = *5.4 Hz, 2 *H*), 2.76–2.74 (m, 2 *H*), 2.41–2.36 (m, 2 *H*), 2.83–1.83 (m, 2 *H*), 2.02–1.96 (m, 2 *H*) (Figure S19); δ_C_ (126 MHz; CDCl_3_) 146.7, 144.9, 141.1, 132.6, 129.9, 124.4, 122.38, 122.19, 114.2, 112.0, 73.3, 63.3, 59.0, 55.8, 49.0, 42.0, 35.9, 27.0, 25.9, 25.0 (Figure S20); **^1^**H –^13^C HSQC (Figure S21) spectrum also supplied; HRMS (ESI) 346.14711 ([M+H]^+^), calcd. for C_19_H_24_NO_3_S^+^ 346.14714; Anal. Calcd. for C_19_H_23_NO_3_S: C, 66.06; H, 6.71; N, 4.05. Found: C, 65.88; H, 6.91; N, 3.87.

### 1-(4-Chlorobenzyl)-6′,7′-dihydrospiro[piperidine-4,4′-thieno[3,2-*c*]pyran] 13

Prepared according to general procedure 1. Following purification by flash chromatography (3∶100∶0.3, CH_3_OH∶CHCl_3_∶NH_4_OH), *the title compound* was obtained as a colourless oil that solidified when taken to 4°C (79 mg, 64%); mp 106–109°C; ν_max_ (film)/cm^−1^ 2923, 2816, 1489, 1076, 1016, 854, 646; δ_H_ (500 MHz; CDCl_3_) 7.29 (s_app_, 4 *H*), 7.07 (d, *J = *5.2 Hz, 1 *H*), 6.81 (d, *J = *5.2 Hz, 1 *H*), 3.92 (t_app_, *J = *5.4 Hz, 2 *H*), 3.52 (s, 2 *H*), 2.82 (t_app_, *J = *5.4 Hz, 2 *H*), 2.70–2.67 (m, 2 *H*), 2.42–2.36 (m, 2 *H*), 1.99–1.93 (m, 2 *H*), 1.86–1.83 (m, 2 *H*) (Figure S22); δ_C_ (126 MHz; CDCl_3_) 141.2, 137.3, 132.8, 130.6, 128.5, 124.5, 122.4, 59.1, 49.2, 36.3, 26.0 (Figure S23); HRMS (ESI) 334.10268 ([M+H]^+^), calcd. for C_18_H_21_ClNOS^+^ 334.10269; Anal. Calcd. for C_18_H_20_ClNOS: C, 64.75; H, 6.04; N, 4.20. Found: C, 64.56; H, 6.08; N, 4.16.

### 1-((1-(4-Fluorophenyl)-2,5-dimethyl-1*H*-pyrrol-3-yl)methyl)-6′,7′- dihydrospiro[piperidine-4,4′-thieno[3,2-*c*]pyran] hydrochloride 14

Prepared according to general procedure 1. Following purification by flash chromatography (2∶100∶0.2, CH_3_OH∶CHCl_3_∶NH_4_OH), *the title compound* was obtained as a brown solid (23 mg, 22%); ν_max_ (film)/cm^−1^ 2922, 1511, 1224, 1075, 852; δ_H_ (500 MHz; CDCl_3_) 7.18–7.14 (m, 3 *H*), 6.88 (s, 1 *H*), 6.02 (s, 1 *H*), 3.93 (t_app_, *J = *5.4 Hz, 2 *H*), 3.57 (brs, 2 *H*), 2.97 (brs, 1 H), 2.83 (t_app_, *J = *5.3 Hz, 2 H), 2.54 (s, 2 H), 2.23 (s, 1 H), 1.99 (s, 4 H), 1.91–1.88 (m, 3 H), 1.68 (s, 3 H) (Figure S24); δ_C_ (126 MHz; CDCl_3_) 163.0, 161.0, 140.6, 135.0, 135.0, 132.7, 130.1, 130.1, 124.7, 122.6, 116.3, 116.1, 108.6, 72.9, 59.4, 54.4, 48.5, 35.3, 29.8, 26.0, 12.9, 11.1 (Figure S25); δ_F_ (471 MHz; CDCl_3_) *-*113.8; HRMS (ESI) 411.19012 ([M−Cl]^+^), calcd. for C_24_H_28_FN_2_OS 411.19009; Anal. Calcd. for C_24_H_28_ClFN_2_OS: C, 64.49; H, 6.31; N, 6.27. Found: C, 64.05; H, 6.14; N, 5.95.

### 1-Cyclohexyl-6′,7′-dihydrospiro[piperidine-4,4′-thieno[3,2-*c*]pyran] 15

Prepared according to general procedure 1. Following purification by flash chromatography (0.8∶100∶0.08, CH_3_OH∶CHCl_3_∶NH_4_OH) *the title compound* was obtained as a colourless solid (67 mg, 84%); mp 170–172°C; ν_max_ (film)/cm^−1^ 2925, 2854, 1076, 647; δ_H_ (500 MHz; CDCl_3_) 7.05 (d, *J = *5.2 Hz, 1 *H*), 6.84 (d, *J = *5.2 Hz, 1 *H*), 3.92 (t, *J = *5.4 Hz, 2 *H*), 2.82 (t, *J = *5.4 Hz, 2 *H*), 2.78–2.76 (m, 2 *H*), 2.65 (t, *J = *11.6 Hz, 2 *H*), 2.38–2.34 (m, 1 *H*), 2.04–1.81 (m, 8 *H*), 1.66–1.62 (m, 1 *H*), 1.32–1.21 (m, 4 *H*), 1.15–1.08 (m, 1 *H*) (Figure S26); δ_C_ (126 MHz; CDCl_3_) 141.2, 132.7, 124.7, 122.3, 73.6, 64.3, 59.1, 44.8, 36.5, 29.0, 26.5, 26.2, 26.0 (Figure S27); HRMS (ESI) 292.17301 ([M+H]^+^), calcd. for C_17_H_26_NOS^+^ 292.17296; Anal. Calcd. for C_17_H_25_NOS: C, 70.06; H, 8.65; N, 4.81. Found: C, 69.51; H, 8.67; N, 4.70. Discrepancy in CHN analysis noted, but data consistent with 5M + H_2_O i.e. calcd. for C_85_H_127_N_5_O_6_S_5_: C, 69.20; H, 8.68; N, 4.75.

### 6′,7′-Dihydrospiro[piperidine-4,4′-thieno[3,2-c]pyran]-1-yl(phenyl) methanone 16

Prepared according to general procedure 2. Following purification by flash chromatography (1∶100∶0.1, CH_3_OH∶CHCl_3_∶NH_4_OH), *the title compound* was obtained as a colourless solid (60 mg, 66%); mp 170–172°C; ν_max_ (film)/cm^−1^ 2951, 1432, 1050, 709; δ_H_ (500 MHz; CDCl_3_) 7.45–7.40 (m, 5 *H*), 7.10 (d, *J = *5.2 Hz, 1 *H*), 6.76 (d, *J = *5.2 Hz, 1 *H*), 4.67–4.64 (m, 1 *H*), 3.97–3.94 (m, 2 *H*), 3.65–3.60 (m, 1 H), 3.50–3.45 (m, 1 *H*), 3.22–3.16 (m, 1 *H*), 2.85 (t, *J = *5.4 Hz, 2 *H*), 2.01–1.75 (m, 4 *H*) (Figure S28); δ_C_ (126 MHz; CDCl_3_; 300 K) 170.5, 140.1, 136.4, 133.0, 129.7, 128.6, 127.1, 124.1, 122.9, 73.5, 59.5, 25.9 (Figure S29); δ_C_ (126 MHz; CDCl_3_; 270 K) 170.5, 139.9, 136.0, 133.0, 129.7, 128.6, 127.0, 124.1, 122.9, 73.4, 59.5, 43.7, 38.1, 36.7, 35.5, 25.8; HRMS (ESI) 336.10287 ([M+Na]^+^), calcd. for C_18_H_19_NO_2_SNa^+^ 336.10287; Anal. Calcd. for C_18_H_19_NO_2_S: C, 68.98; H, 6.11; N, 4.47. Found: C, 68.03; H, 6.05; N, 4.44. Discrepancy in CHN analysis noted, but data consistent with 4M + H_2_O i.e. calcd. for C_72_H_78_N_4_O_9_S_4_: C, 68.00; H, 6.18; N, 4.41. ^13^C{^1^H} signals are missing or obscured due to rotamers around the amide bond. The signals were visualised using **^1^**H –^13^C HSQC spectroscopy (Figure S30) and completely resolved at 270 K (Figure S40). Variable temperature NMR data for this compound may be found in Dataset S1.

### (4-Bromophenyl)(6′,7′-dihydrospiro[piperidine-4,4′-thieno[3,2-c]pyran]-1-yl) methanone 17

Prepared according to general procedure 2. Following purification by flash chromatography (1∶100∶0.1, CH_3_OH∶CHCl_3_∶NH_4_OH), *the title compound* was obtained as a colourless solid (60 mg, 66%); mp 170–172°C; ν_max_ (film)/cm^−1^ 2923, 1628, 1433, 1072; δ_H_ (500 MHz; CDCl_3_) 7.56–7.52 (m, 2 *H*), 7.39–7.30 (m, 2 *H*), 7.10 (d, *J* = 5.2 Hz, 1 *H*), 6.74 (d, *J* = 5.2 Hz, 1 *H*), 4.65–4.58 (m, 1 *H*), 3.95–3.92 (m, 2 *H*), 3.62–3.44 (m, 2 *H*), 3.23–3.15 (m, 1 *H*), 2.85 (t_app_, *J* = 5.4 Hz, 2 *H*), 2.02–1.83 (m, 2 *H*), 1.83–1.72 (m, 2 *H*) (Figure S31); δ_C_ (126 MHz; CDCl_3_) 169.5, 139.9, 135.1, 133.1, 131.8, 128.8, 124.00, 123.97, 122.9, 73.4, 59.5, 25.9 (Figure S32); **^1^**H –^13^C HSQC (Figure S33) and ^1^H –^13^C{^1^H} HMBC (Figure S34) spectra also supplied; HRMS (ESI) 416.01134 ([M+Na]^+^), calcd. for C_18_H_18_^81^BrNOSNa^+^ 416.01134; Anal. Calcd. for C_18_H_18_BrNO_2_S: C, 55.11; H, 4.62; N, 3.57. Found: C, 55.88; H, 4.89; N, 3.45. ^13^C{^1^H} signals are missing or obscured due to rotamers around the amide bond. Discrepancy in CHN analysis noted; data match sample containing <10% des-brominated compound, but this was not observed in the ^1^H NMR spectrum for this sample.

### (2-Bromophenyl)(6′,7′-dihydrospiro[piperidine-4,4′-thieno[3,2-c]pyran]-1-yl) methanone 18

Prepared according to general procedure 2. Following purification by flash chromatography (1∶100∶0.1, CH_3_OH∶CH_2_Cl_2_∶NH_4_OH), *the title compound* was obtained as a colourless solid (81 mg, 73%); mp 48–52°C; ν_max_ (film)/cm^−1^ 2932, 1634, 1436, 1073, 768; δ*_H_* (500 MHz; CDCl_3_) 7.60–7.54 (m, 1 *H*), 7.38–7.31 (m, 2 *H*), 7.24 (dd, *J = *13.3, 6.7 Hz, 2 *H*), 7.08 (dd, *J* = 4.8, 2.5 Hz, 1 *H*), 6.72 (d, *J* = 5.2 Hz, 1 *H*), 4.73–4.66 (m, 1 *H*), 3.93 (tq, *J* = 10.0, 4.9 Hz, 2 *H*), 3.55 (td, *J = *12.9, 3.2 Hz,), 3.42–3.34 (m, 1 *H*), 3.28–3.12 (m, 2 *H*), 2.83 (t, *J = *4.9 Hz, 2 *H*), 2.10 (td, *J* = 13.5, 5.0 Hz, 1 *H*), 1.99 (dd, *J* = 9.8, 3.6 Hz, 1 *H*), 1.94–1.90 (m, 1 *H*), 1.81–1.69 (m, 2 *H*), 1.26 (t, *J = *7.2 Hz, 1 *H*), 1.05 (t, *J = *7.1 Hz, <1 *H*) (Figure S35); δ_C_ (126 MHz; CDCl_3_) 167.8, 167.6, 140.0, 139.9, 138.9, 138.5, 138.4, 133.4, 132.9, 132.8, 132.8, 130.3, 130.2, 130.0, 127.8, 127.7, 127.7, 127.6, 127.6, 127.5, 124.1, 123.9, 122.8, 122.8, 119.4, 119.2, 73.4, 73.3, 59.5, 43.5, 42.8, 42.5, 39.0, 37.7, 37.5, 36.6, 36.3, 35.6, 35.5, 31.0, 25.8, 14.0, 12.6 (Figure S36); **^1^**H –^13^C HSQC (Figure S37) and ^1^H –^13^C{^1^H} HMBC (Figure S38) spectra also supplied; HRMS (ESI) 414.01338 ([M+Na]^+^), calcd. for C_18_H_18_BrNO_2_SNa^+^ 414.01338; Anal. Calcd. for C_18_H_18_BrNO_2_S: C, 55.11; H, 4.62; N, 3.57. Found: C, 55.36; H, 4.36; N, 3.59. The additional ^13^C{^1^H} and ^1^H signals are due to rotamers around the amide bond.

### Resazurin assay of growth inhibition

Compounds were tested for activity at either single concentration (100 µM) or serially diluted in 10 µL of purified H_2_O in triplicate in 96 well microtiter plates. *M. tuberculosis* H37Rv was grown in complete Middlebrook 7H9 media (Bacto, Australia) containing albumin, dextrose and catalase (ADC), 20% Tween 80 and 50% glycerol (Sigma-Aldrich, Australia). A bacterial suspension (90 µL) at OD_600nm_ of 0.001 was added to the wells and incubated for 7 days. Resazurin (10 µL; 0.05%(w/v); Sigma-Aldrich, Australia) was then added, incubated for 24 h at 37°C, and fluorescence measured at 590 nm using a FLUOstar Omega microplate reader (BMG Labtech, Germany). After subtraction of background fluorescence from all wells, the percentage mycobacterial survival was determined by comparing the fluorescence of wells containing compounds compared to control wells not treated with compound.

### Compound intracellular efficacy and toxicity

THP1 cells (TIB-202R), a human monocyte cell line (American Type Culture Collection, USA), were grown in complete Dulbecco's Modified Eagle Media (DMEM; LifeTechnologies, Australia) including 10% fetal bovine serum (FBS), 200 µM L-glutamine (LifeTechnologies, Australia), and 1 mM HEPES buffer solution (LifeTechnologies, Australia). Cells (1×10^5^) in media containing 50 ng/mL phorbol 12-myristate 13-acetate (PMA) were added to 96-well plates and were then incubated for 48 h at 37°C to allow adherence and differentiation. Compounds (1.56–200 µM) were added to the wells and incubated for 4 days at 37°C. Then 0.05%(w/v) resazurin (4 h) was added and the fluorescence measured. Cell viability was calculated as percentage fluorescence in comparison to untreated cells.

## Supporting Information

Figure S1 ^1^H NMR (500 MHz, CDCl_3_) spectrum of 6′,7′-dihydrospiro[piperidine-4,4′-thieno[3,2-*c*]pyran] **3**.
(PDF)

Figure S2 ^13^C{^1^H} NMR (126 MHz, CDCl_3_) spectrum of 6′,7′-dihydrospiro[piperidine-4,4′-thieno[3,2-*c*]pyran] **3**.
(EPS)

Figure S3 **^1^**H –^13^C HSQC (500 MHz, CDCl_3_) spectrum of 6′,7′-dihydrospiro[piperidine-4,4′-thieno[3,2-*c*]pyran] **3**.
(EPS)

Figure S4 ^1^H –^13^C{^1^H} HMBC (500 MHz, CDCl_3_) spectrum of 6′,7′-dihydrospiro[piperidine-4,4′-thieno[3,2-*c*]pyran] **3**.
(EPS)

Figure S5 ^1^H NMR (500 MHz, CDCl_3_) spectrum of 1-benzyl-6′,7′-dihydrospiro[piperidine-4,4′-thieno[3,2-*c*]pyran] **6**.
(PDF)

Figure S6 ^13^C{^1^H} NMR (126 MHz, CDCl_3_) spectrum of 1-benzyl-6′,7′-dihydrospiro[piperidine-4,4′-thieno[3,2-*c*]pyran] **6**.
(EPS)

Figure S7 **^1^**H –^13^C HSQC (500 MHz, CDCl_3_) spectrum of 1-benzyl-6′,7′-dihydrospiro[piperidine-4,4′-thieno[3,2-*c*]pyran] **6**.
(EPS)

Figure S8 ^1^H –^13^C{^1^H} HMBC (500 MHz, CDCl_3_) spectrum of 1-benzyl-6′,7′-dihydrospiro[piperidine-4,4′-thieno[3,2-*c*]pyran] **6**.
(EPS)

Figure S9 **^1^**H NMR (500 MHz, CDCl_3_) spectrum of 2-chloroethyl 6′,7′-dihydrospiro[piperidine-4,4′-thieno[3,2-*c*]pyran]-1-carboxylate **9**.
(EPS)

Figure S10 **^13^**C{^1^H} NMR (126 MHz, CDCl_3_) spectrum of 2-chloroethyl 6′,7′-dihydrospiro[piperidine-4,4′-thieno[3,2-*c*]pyran]-1-carboxylate **9**.
(EPS)

Figure S11 **^1^**H –^13^C HSQC (500 MHz, CDCl_3_) spectrum of 2-chloroethyl 6′,7′-dihydrospiro[piperidine-4,4′-thieno[3,2-*c*]pyran]-1-carboxylate **9**.
(EPS)

Figure S12 ^1^H –^13^C{^1^H} HMBC (500 MHz, CDCl_3_) spectrum of 2-chloroethyl 6′,7′-dihydrospiro[piperidine-4,4′-thieno[3,2-*c*]pyran]-1-carboxylate **9**.
(EPS)

Figure S13 ^1^H NMR (500 MHz, CDCl_3_) spectrum of 1-((2,3-dihydrobenzo[*b*][1], [4]dioxin-6-yl)methyl)-6′,7′-dihydrospiro[piperidine- 4,4′-thieno[3,2-*c*]pyran] **10**.
(EPS)

Figure S14 ^13^C{^1^H} NMR (126 MHz, CDCl_3_) spectrum of 1-((2,3-dihydrobenzo[*b*][1], [4]dioxin-6-yl)methyl)-6′,7′-dihydrospiro[piperidine- 4,4′-thieno[3,2-*c*]pyran] **10**.
(EPS)

Figure S15 **^1^**H –^13^C HSQC (500 MHz, CDCl_3_) spectrum of 1-((2,3-dihydrobenzo[*b*][1], [4]dioxin-6-yl)methyl)-6′,7′-dihydrospiro[piperidine- 4,4′-thieno[3,2-*c*]pyran] **10**.
(EPS)

Figure S16 ^1^H –^13^C{^1^H} HMBC (500 MHz, CDCl_3_) spectrum of 1-((2,3-dihydrobenzo[*b*][1], [4]dioxin-6-yl)methyl)-6′,7′-dihydrospiro[piperidine- 4,4′-thieno[3,2-*c*]pyran] **10**.
(EPS)

Figure S17 **^1^**H NMR (500 MHz, CDCl_3_) spectrum of 1-(benzo[*d*][1], [3]dioxin-5-ylmethyl)-6′,7′-dihydrospiro[piperidine- 4,4′-thieno [3,2-*c*]pyran] **11**.
(EPS)

Figure S18 **^13^**C{^1^H} NMR (126 MHz, CDCl_3_) spectrum of 1-(benzo[*d*][1], [3]dioxin-5-ylmethyl)-6′,7′-dihydrospiro[piperidine- 4,4′-thieno [3,2-*c*]pyran] **11**.
(EPS)

Figure S19 ^1^H NMR (500 MHz, CDCl_3_) spectrum of 4-((6′,7′-dihydrospiro[piperidine-4,4′-thieno[3,2-*c*]pyran]-1-yl) (2-methoxyphenol) **12**.
(EPS)

Figure S20 **^13^**C{^1^H} NMR (126 MHz, CDCl_3_) spectrum of 4-((6′,7′-dihydrospiro[piperidine-4,4′-thieno[3,2-*c*]pyran]-1-yl) (2-methoxyphenol) **12**.
(EPS)

Figure S21 **^1^**H –^13^C HSQC (500 MHz, CDCl_3_) spectrum of 4-((6′,7′-dihydrospiro[piperidine-4,4′-thieno[3,2-*c*]pyran]-1-yl) (2-methoxyphenol) **12**.
(EPS)

Figure S22 ^1^H NMR (500 MHz, CDCl_3_) spectrum of 1-(4-chlorobenzyl)-6′,7′-dihydrospiro[piperidine-4,4′-thieno[3,2-*c*]pyran] **13**.
(EPS)

Figure S23 ^13^C{^1^H} NMR (126 MHz, CDCl_3_) spectrum of 1-(4-chlorobenzyl)-6′,7′-dihydrospiro[piperidine-4,4′-thieno[3,2-*c*]pyran] **13**.
(EPS)

Figure S24 ^1^H NMR (500 MHz, CDCl_3_) spectrum of 1-((1-(4-fluorophenyl)-2,5-dimethyl-1*H*-pyrrol-3-yl)methyl)-6′,7′- dihydrospiro[piperidine-4,4′-thieno[3,2-*c*]pyran] hydrochloride **14**.
(EPS)

Figure S25 ^13^C{^1^H} NMR (126 MHz, CDCl_3_) spectrum of 1-((1-(4-fluorophenyl)-2,5-dimethyl-1*H*-pyrrol-3-yl)methyl)-6′,7′- dihydrospiro[piperidine-4,4′-thieno[3,2-*c*]pyran] hydrochloride **14**.
(PDF)

Figure S26 ^1^H NMR (500 MHz, CDCl_3_) spectrum of 1-cyclohexyl-6′,7′-dihydrospiro[piperidine-4,4′-thieno[3,2-*c*]pyran] **15**.
(EPS)

Figure S27 ^13^C{^1^H} NMR (126 MHz, CDCl_3_) spectrum of 1-cyclohexyl-6′,7′-dihydrospiro[piperidine-4,4′-thieno[3,2-*c*]pyran] **15**.
(EPS)

Figure S28 ^1^H NMR (500 MHz, CDCl_3_) spectrum of 6′,7′-dihydrospiro[piperidine-4,4′-thieno[3,2-c]pyran]-1-yl(phenyl) methanone **16**.
(EPS)

Figure S29 ^13^C{^1^H} NMR (126 MHz, CDCl_3_) spectrum of 6′,7′-dihydrospiro[piperidine-4,4′-thieno[3,2-c]pyran]-1-yl(phenyl) methanone **16**.
(EPS)

Figure S30 **^1^**H –^13^C HSQC (500 MHz, CDCl_3_) spectrum of 6′,7′-dihydrospiro[piperidine-4,4′-thieno[3,2-c]pyran]-1-yl(phenyl) methanone **16**.
(EPS)

Figure S31 ^1^H NMR (500 MHz, CDCl_3_) spectrum of (4-bromophenyl)(6′,7′-dihydrospiro[piperidine-4,4′-thieno[3,2-c]pyran]-1-yl) methanone **17**.
(EPS)

Figure S32 ^13^C{^1^H} NMR (126 MHz, CDCl_3_) spectrum of (4-bromophenyl)(6′,7′-dihydrospiro[piperidine-4,4′-thieno[3,2-c]pyran]-1-yl) methanone **17**.
(EPS)

Figure S33 **^1^**H –^13^C HSQC (500 MHz, CDCl_3_) spectrum of (4-bromophenyl)(6′,7′-dihydrospiro[piperidine-4,4′-thieno[3,2-c]pyran]-1-yl) methanone **17**.
(EPS)

Figure S34 ^1^H –^13^C{^1^H} HMBC (500 MHz, CDCl_3_) spectrum of (4-bromophenyl)(6′,7′-dihydrospiro[piperidine-4,4′-thieno[3,2-c]pyran]-1-yl) methanone **17**.
(EPS)

Figure S35 ^1^H NMR (500 MHz, CDCl_3_) spectrum of (2-bromophenyl)(6′,7′-dihydrospiro[piperidine-4,4′-thieno[3,2-c]pyran]-1-yl) methanone **18**.
(EPS)

Figure S36 ^13^C{^1^H} NMR (126 MHz, CDCl_3_) spectrum of (2-bromophenyl)(6′,7′-dihydrospiro[piperidine-4,4′-thieno[3,2-c]pyran]-1-yl) methanone **18**.
(EPS)

Figure S37 **^1^**H –^13^C HSQC (500 MHz, CDCl_3_) spectrum of (2-bromophenyl)(6′,7′-dihydrospiro[piperidine-4,4′-thieno[3,2-c]pyran]-1-yl) methanone **18**.
(EPS)

Figure S38 ^1^H –^13^C{^1^H} HMBC (500 MHz, CDCl_3_) spectrum of (2-bromophenyl)(6′,7′-dihydrospiro[piperidine-4,4′-thieno[3,2-c]pyran]-1-yl) methanone **18**.
(EPS)

Figure S39 ^1^H NMR (CDCl_3_) spectra (4.5–2.5 ppm) of 6′,7′-dihydrospiro[piperidine-4,4′-thieno[3,2-c]pyran]-1-yl(phenyl) methanone **16** at variable temperature and magnetic field strength.
(EPS)

Figure S40 Overlay of the ^13^C{^1^H} NMR (126 MHz, CDCl_3_) spectra of 6′,7′-dihydrospiro[piperidine-4,4′-thieno[3,2-c]pyran]-1-yl(phenyl) methanone **16** at 300 K and 270 K.
(EPS)

Text S1 **General synthetic and chemical analysis methods.**
(RTF)

Spreadsheet S1 **Summary of the experimental the experimental data.**
(XLSX)

Dataset S1 Variable Temperature NMR Data for Compound **16**.
(ZIP)
