# Supplementary figures and images for: Efficient Synthesis and Anti-Tubercular Activity of a Series of Spirocycles: An Exercise in Open Science
Source: PLoS One. 2014 Dec 10;9(12):e111782. doi: 10.1371/journal.pone.0111782 (PMC4262224; doi:10.1371/journal.pone.0111782)

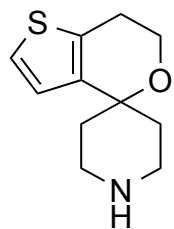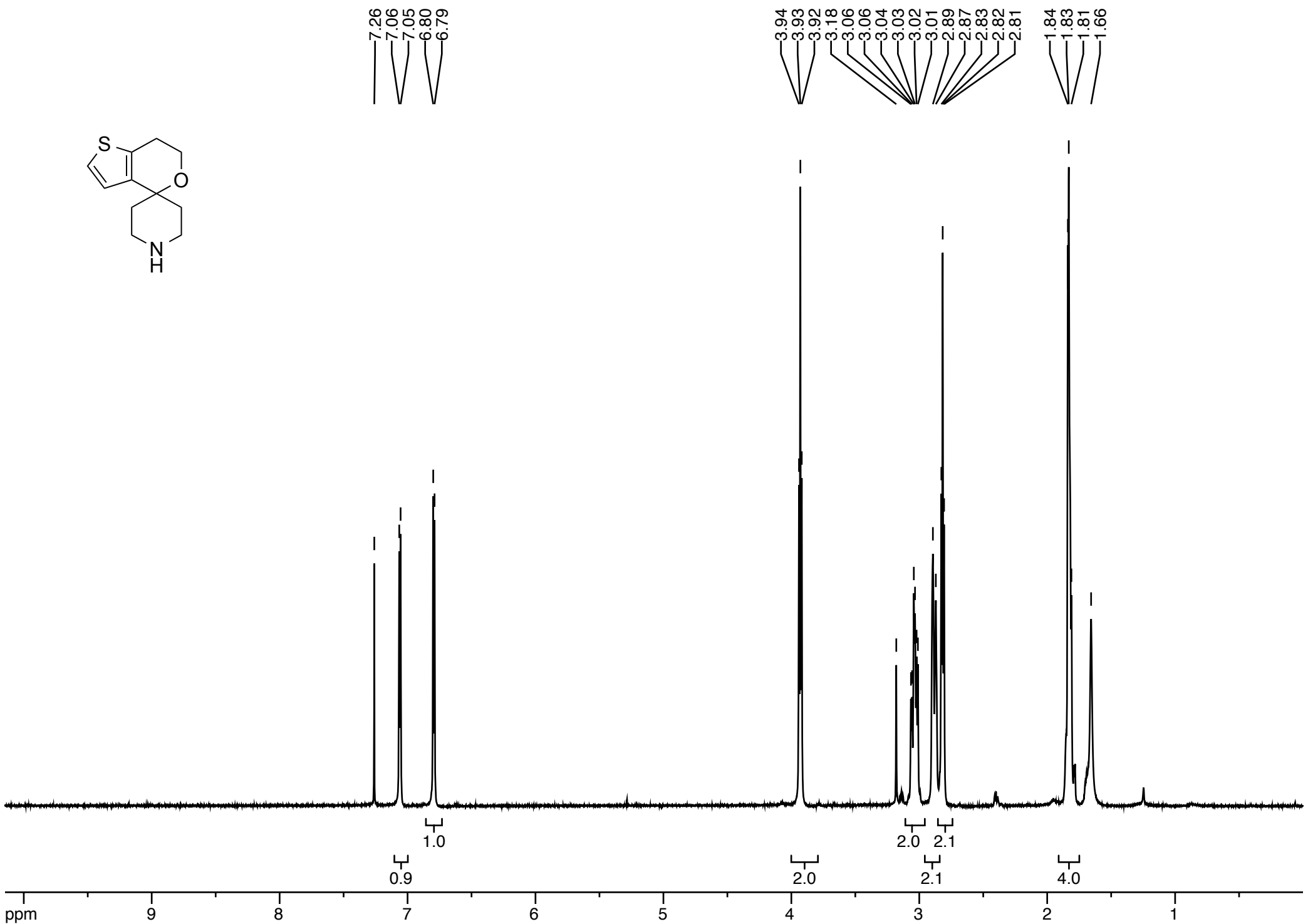

Supplement: Figure S1 — 1H NMR (500 MHz, CDCl3) spectrum of 6′,7′-dihydrospiro[piperidine-4,4′-thieno[3,2-c]pyran] 3. (PDF) [file pone.0111782.s001.pdf]

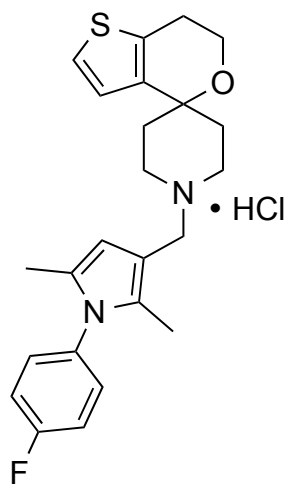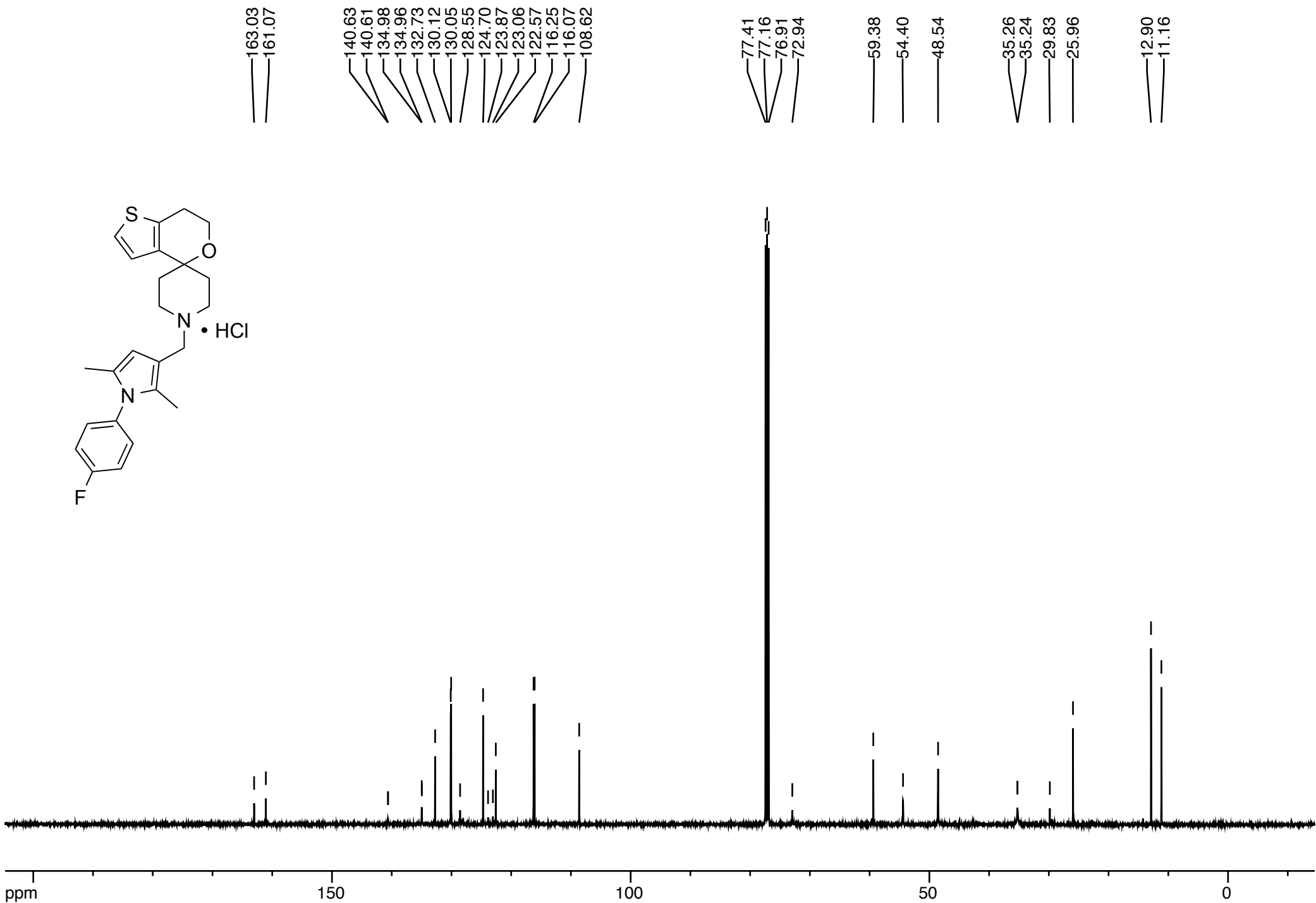

Supplement: Figure S25 — 13C{1H} NMR (126 MHz, CDCl3) spectrum of 1-((1-(4-fluorophenyl)-2,5-dimethyl-1H-pyrrol-3-yl)methyl)-6′,7′- dihydrospiro[piperidine-4,4′-thieno[3,2-c]pyran] hydrochloride 14. (PDF) [file pone.0111782.s025.pdf]

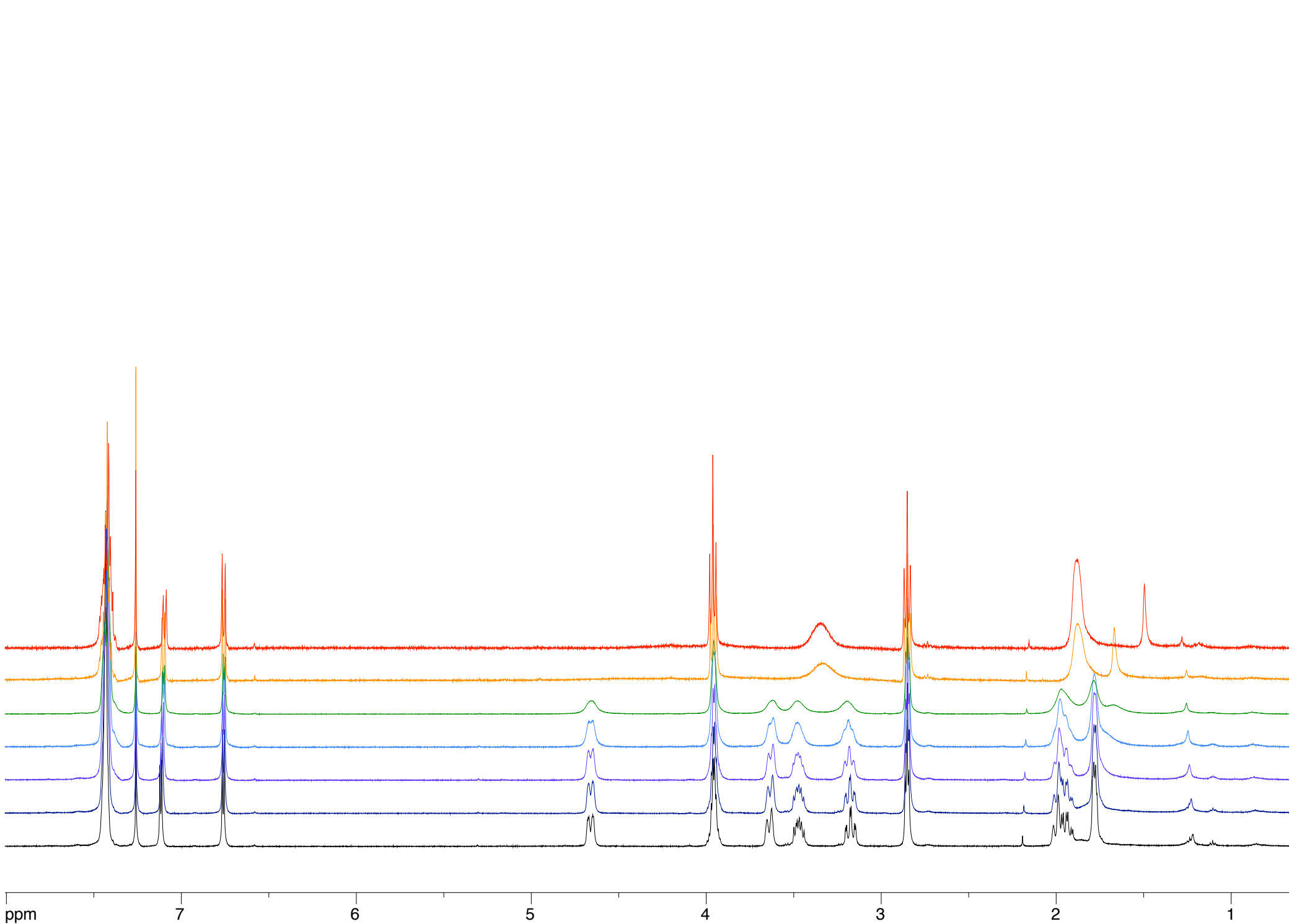

Supplement: Dataset S1 — Variable Temperature NMR Data for Compound 16. (ZIP) [file pone.0111782.s043.zip › NMR300 KAB112-1/2/alloverlay.pdf]
