# Supplementary material for: Efficient Synthesis and Anti-Tubercular Activity of a Series of Spirocycles: An Exercise in Open Science
Source: PLoS One. 2014 Dec 10;9(12):e111782. doi: 10.1371/journal.pone.0111782 (PMC4262224; doi:10.1371/journal.pone.0111782)

Current Data Parameters
NAME KAB90-2
EXPNO 1
PROCNO 1

F2 - Acquisition Parameters
Date_ 20130616
Time 13.26
INSTRUM spect
PROBHD 5 mm PABBO BB-
PULPROG zg
TD 65536
SOLVENT CDCl3
NS 1
DS 0
SWH 10000.000 Hz
FIDRES 0.152588 Hz
AQ 3.2768500 sec
RG 79.92
DW 50.000 usec
DE 6.50 usec
TE 300.0 K
D1 2.00000000 sec

======== CHANNEL f1 ========
NUC1 1H
P1 12.00 usec
PLW1 15.48799992 W
SFO1 500.1330008 MHz

F2 - Processing parameters
SI 32768
SF 500.1300129 MHz
WDW no
SSB 0
LB 0 Hz
GB 0
PC 1.00

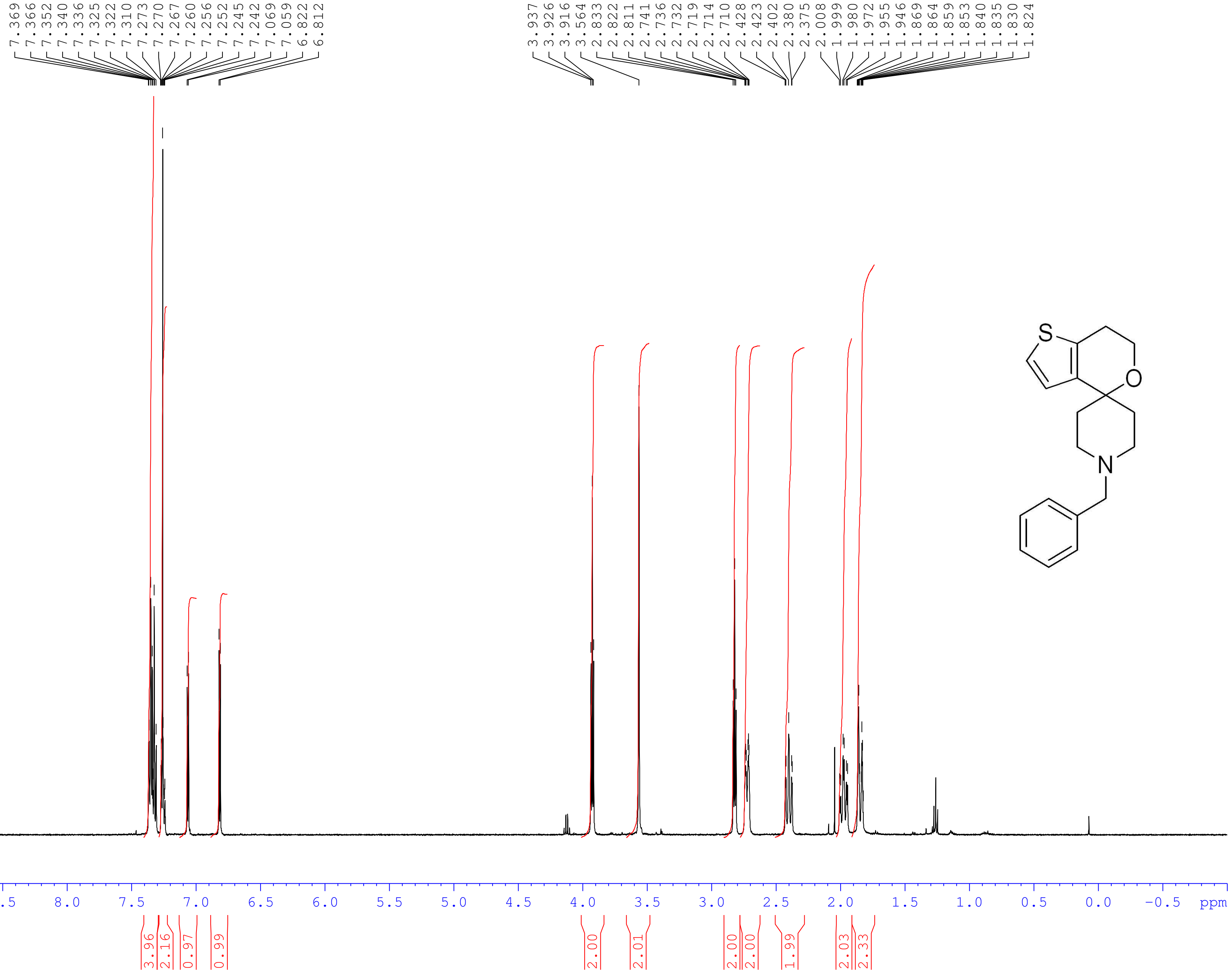

Supplement: Figure S5 — 1H NMR (500 MHz, CDCl3) spectrum of 1-benzyl-6′,7′-dihydrospiro[piperidine-4,4′-thieno[3,2-c]pyran] 6. (PDF) [file pone.0111782.s005.pdf]
