# Supplementary material for: Efficient Synthesis and Anti-Tubercular Activity of a Series of Spirocycles: An Exercise in Open Science
Source: PLoS One. 2014 Dec 10;9(12):e111782. doi: 10.1371/journal.pone.0111782 (PMC4262224; doi:10.1371/journal.pone.0111782)
Supplement: Text S1 — General synthetic and chemical analysis methods. (RTF) [file pone.0111782.s041.rtf]

Reagents were purchased from either Sigma–Aldrich, Alfa Aesar, Acros, Merck, Fischer Scientific or Ajax. Unless otherwise specified, the reagents were used without further purification. For anhydrous conditions, glassware was dried at >130 °C for >12 h, assembled hot and allowed to cool under a high vacuum where appropriate or purged with inert gas. Powdered molecular sieves (MS) were potassium silica-alumina and were microwave activated before use. Anhydrous solvents were dried over microwave activated Ajax 3 Å molecular sieves (1.5–2.5 mm) according to the literature [1]. Nitrogen gas was dried over silica and calcium chloride. Argon and hydrogen gas were used as acquired. In vacuo corresponds to <20 mbar. Reduced pressure means under rotary evaporation at 40 °C from (900–50 mbar) Flash chromatography was performed on Davisil Grace Davison 40–63 mm (230–400 mesh) silica gel. Analytical thin layer chromatography was performed on Merck Silica Gel 60 F254 precoated aluminium plates (0.2 mm) and visualised with UV irradiation (254 nm) and permangenate, anisaldehyde or ninhydrin staining. High temperature reactions were carried out in silicone oil baths, controlled by a temperature probe in the oil bath. Reactions at -78 °C were carried out in dry ice/acetone baths. Melting points (mp) were recorded on a Stanford Research Systems OptiMelt at 1 °C min-1 (capillaries ø = 1.5–1.6 mm, 90 mm). Infrared spectroscopy was carried out on either a Bruker Alpha-E (attenuated total reflectance) or a Bruker Alpha Platinum-ATR without atmospheric compensation and processed using OPUS 6.5 or OPUS 7 software; samples were analysed as thin films following dissolution in acetone and removal of the solvent by evaporation. Nuclear magnetic resonance (NMR) spectroscopy was carried out at 300 K on Bruker spectrometers: either an AVANCE III 400 (1H at 400 MHz, 13C at 101 MHz) or an AVANCE III 500 (1H at 500 MHz, 13C at 126 MHz, 19F at 471 MHz). Spectra were processed using Bruker Topspin or Mestrelab Research iNMR. Deuterated solvents (CDCl3, CDCl3 (0.03% v/v TMS) were obtained from the Cambridge Isotope Laboratories. 1H, 13C and 19F chemical shifts are reported in parts per million (ppm) with respect to TMS at 0.00 ppm. The chemical shifts of the spectra were calibrated to residual solvent peaks (1H: CHCl3 7.26 ppm; 13C: CHCl3 77.16 ppm). 1H signal multiplicity was reported as: s = singlet, d = doublet, t = triplet, dd = doublet of doublets, ddd = doublet of doublet of doublets, and m = multiplet. Broad signals were designated b = broad. Coupling constants (J) are reported in Hertz (Hz). Integrals are relative. app = apparent was  when the multiplicity was unexpected, e.g. coincidental or unresolved. Two-dimensional NMR experiments were carried out following 90° pulse width (P1) calibration and T1 calibration using inversion recovery experiments.
Low-resolution mass spectrometry (m/z) was carried out on a Finnigan quadrupole ion trap mass spectrometer using electrospray ionisation (ESI) or atmospheric-pressure chemical ionisation (APCI). High-resolution mass spectrometry (HRMS) was performed on a Bruker 7T FT-ICR using ESI or APCI. Positive and negative detection is indicated by the charge of the ion, e.g. [M+H]+ indicates positive ion detection. Elemental analyses were carried out at the University of Otago using a Carbo-Erba EA 1108.

[1] DOI: 10.1021/jo101589h
